# Consistent Condom Use Increases the Regression Rate of Cervical Intraepithelial Neoplasia 2–3

**DOI:** 10.1371/journal.pone.0045114

**Published:** 2012-09-13

**Authors:** Ane Cecilie Munk, Einar Gudlaugsson, Anais Malpica, Bent Fiane, Kjell I. Løvslett, Arnold-Jan Kruse, Irene Tveiterås Øvestad, Feja Voorhorst, Emiel A. M. Janssen, Jan P. A. Baak

**Affiliations:** 1 Department of Obstetrics and Gynecology, Stavanger University Hospital, Stavanger, Norway; 2 Department of Pathology, Stavanger University Hospital, Stavanger, Norway; 3 Department of Pathology and Gynecologic Oncology, The University of Texas M. D. Anderson Cancer Center, Houston, Texas, United States of America; 4 Department of Gynecology, Academic Hospital Maastricht, Maastricht, The Netherlands; 5 Department of Epidemiology and Biostatistics, VU Medical Center, Amsterdam, The Netherlands; University of Ottawa, Canada

## Abstract

**Objective:**

Cervical intraepithelial neoplasia grades 2-3 (CIN2-3) are usually treated by cone excision, although only 30% progress to cancer and 6–50% regress spontaneously. The aim of this study was to examine the influence of clinical factors like smoking habits, number of lifetime sexual partners, age at first sexual intercourse, sexual activity span and hormonal versus non-hormonal contraception type on the regression rate of CIN2-3.

**Methods:**

In this prospective population-based cohort study 170 women aged 25–40 with abnormal cytology and colposcopy-directed biopsies showing first time onset CIN2-3 were consecutively included. The interval between biopsy and cone excision was standardized to minimum 12 weeks. Regression was defined as ≤CIN1 in the cone biopsy.

**Results:**

The regression rate was 22%. Consistent condom use, defined as those women whose partners used condoms for all instances of sexual intercourse, was infrequent (n = 20, 12%). In univariate analysis consistent condom use, hormonal contraception and age at first sexual intercourse significantly predicted regression. In a multivariate analysis only consistent condom use remained as an independent predictor of regression (regression rate 55%, p = 0.001, hazard ratio = 4.4).

**Conclusion:**

Consistent condom use between punch biopsy and cone excision in first-time onset CIN2-3 patients significantly increases the regression rate.

## Introduction

Human papillomavirus (HPV) is the major cause of cervical intraepithelial neoplasia (CIN) [1]. CIN lesions are dynamic and can persist or progress to invasive cancer [2], while 6–50% of all CIN2-3 lesions regress spontaneously [3]–[5]. When left untreated, about 31% of CIN3s will progress into invasive squamous carcinoma (often over many years), compared to 0.7% of CIN3s in conventionally treated women [6].

Standard treatment of CIN2-3 is cone excision, an invasive procedure with potential complications [7]. Cervical insufficiency is the most serious late-complication potentially leading to late abortion or preterm delivery [8], [9]. Since the large majority of patients treated with cone excision are fertile women, the risk of cervical insufficiency is a major concern [10].

The natural history of CIN-lesions may be influenced by the HPV genotypes, the individual general and local immune response and epithelial factors. Sexual behaviour, parity, contraceptives, smoking habits and genetics are widely reported risk factors for CIN development [5], [11]–[17], but only a few studies have examined the influence of these clinical factors on regression of already established CIN lesion [5], [18], [19].

In order to evaluate the results from these hypothesis-generating studies, we performed a prospective validation study investigating the influence of hormonal contraception, number of lifetime sexual partners, age at first sexual intercourse, smoking habits, CIN-grade 2 or −3, age at diagnosis, biopsy–cone interval, parity and family history of CIN or cervical cancer on the regression rate in women with histological proven first-time onset CIN2-3. As Hogewoning et al. reported that condom use promotes regression of CIN2-3 and clearance of HPV in a prospective randomized trial, special attention was given to validate the effect of condom use on the regression rate [18].

It has previously been shown that an interval less than 9 weeks is associated with a low regression rate of 5%. The regression rate was extensively higher (38%), when intervals exceeded 9 weeks [20]. Hogewoning et al also showed that the longer the interval, the higher the regression rate [18]. Therefore, the interval between biopsy and cone excision was limited to 12 weeks and more in the current study.

## Materials and Methods

The study was approved by the Norwegian Regional Ethical committee (# 303.06), the Social and Health Department (#07/3300) and the Norwegian Social Science Data Service (#17185). All patients gave written informed consent. The age was limited to <41 years as the Ethics Committee regarded the risk of developing micro invasive carcinoma in patients more than 40 years as too high.

### Gynecologic Methods

Women aged 25–40 years, who were referred to the gynecology outpatient clinic, Stavanger University Hospital, between January 2007 and December 2008, for further diagnostic evaluation of a population screening detected atypical cytological cervical smear, were asked to participate. Atypical cytology was defined according to the national Norwegian guidelines as: 1. LSIL + high-risk (hr)-HPV; 2. ASCUS + hr-HPV; 3. Recurrences of two hr-HPV positive cervical smears within six months; 4. ASC-H or 5. HSIL.

**Figure 1:**
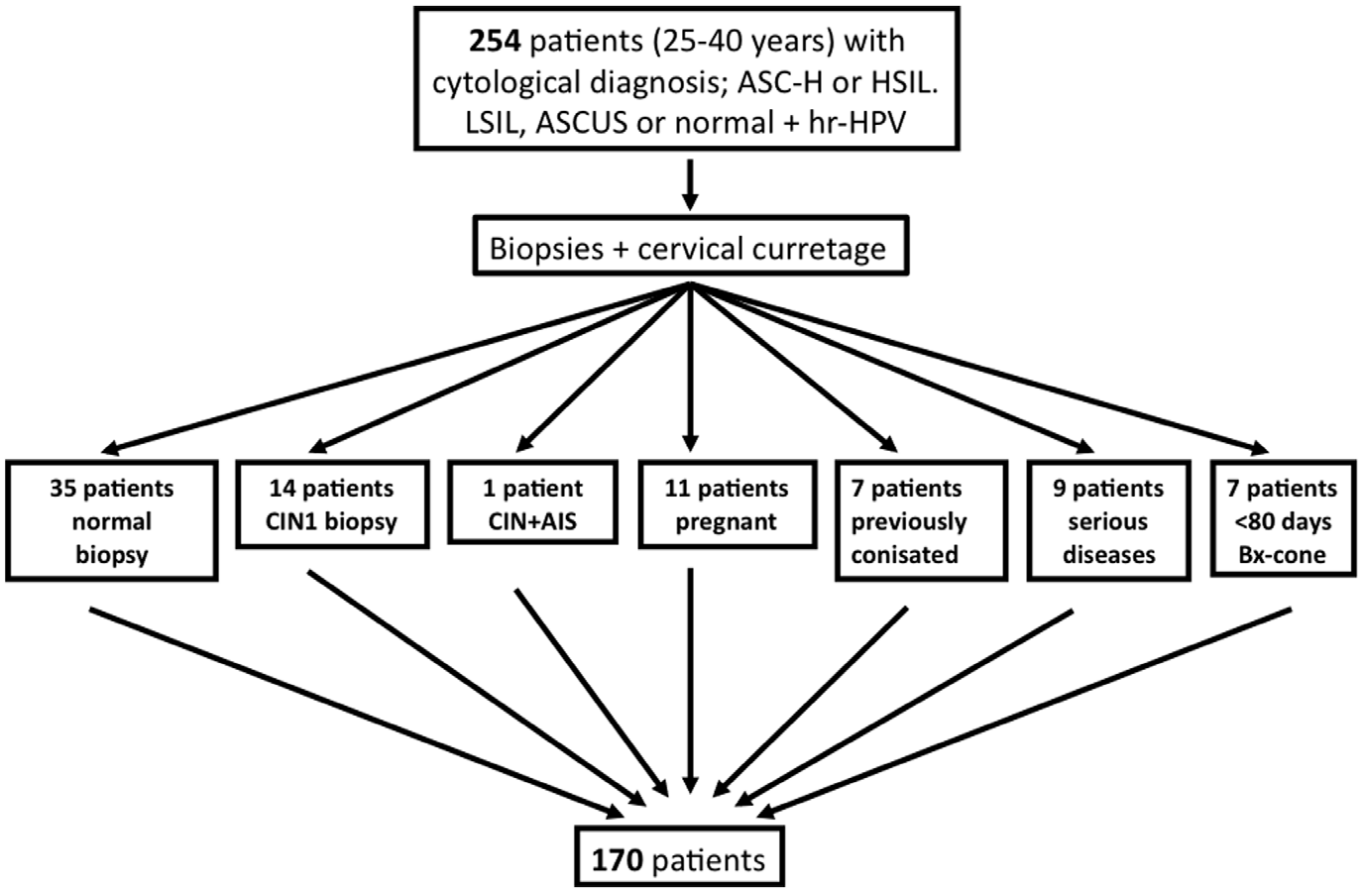
Inclusion and exclusion criteria. Norwegian guidelines for histological examination of atypical cytology were followed: 1. Low grade Squamous Intraepithelial Lesion (LSIL) + high-risk Human Papillomavirus (hr-HPV) or 2. Atypical Squamous Cells of Undetermined Significance (ASCUS) + hr-HPV or 3. Recurrences of hr-HPV positive cervical smears or 4. Atypical Squamous Cells, cannot rule out a High Grade lesion (ASC-H) or 5. High Grade Squamous Intrepithelial Lesion (HSIL). AIS denotes Adenocarcinoma in situ and Bx Biopsy.

Of the 254 initially included patients, 84 patients were excluded due to normal or CIN1 histology in the biopsy, pregnancy, significant comorbidity defined as diseases affecting the immune system or need of treatment with immunosuppressive drugs, previous treatment for CIN and biopsy-cone excision intervals shorter than 80 days (Figure 1).

As part of the standard follow-up at the gynecology outpatient clinic, Stavanger University Hospital, the patients came for colposcopy, biopsy and endocervical curettage followed by cone excision, if biopsy revealed CIN2 or CIN3. An additional visit 7–9 weeks after biopsy was part of the study protocol, due to the slightly longer interval between biopsy and cone excision. At this visit colposcopy was performed to detect eventually fast developing premalignant mucosal changes.

Follow-up consisted of at least 3 visits to the gynecology outpatient clinic as follows:

At the 1^st^ visit, clinical data including sexual history, contraception type, smoking habits, parity and family history of cervical dysplasia or cancer were registered. The patients were asked to continue their actual contraception. Colposcopy was performed prior to punch biopsies and endocervical curettage.

At the 2^nd^ visit (week 7–9) a second colposcopy was performed.

At the 3^rd^ visit (between 12 and 24 weeks) information about eventual changes of contraception type, including condom use was collected. Consistent condom use was defined as those women, whose partners used condoms for all instances of sexual intercourse (in contrast to inconsistent condom use). Finally, after a third colposcopy, a cone excision of the abnormal area was performed using Loop Electrosurgical Excision Procedure.

Colposcopy at punch biopsy and cone excision is standard in our hospital, and all colposcopy findings were documented. Biopsy–cone excision intervals exceeding 16 weeks were patient related. All patients were treated according to Norwegian guidelines.

### Pathology

Punch biopsies and cone biopsy material were fixed in 4% buffered formaldehyde (24–48 hours) at ∼20°C and embedded in 56°C paraffin. Eosin staining was performed prior to embedding of the biopsies, to allow macroscopic identification of the epithelium and reduce tangential cutting. Standard Hematoxylin Erythrosin Saffran sections were used for histopathological evaluation. As the diagnosis of CIN-1, 2 and 3, metaplasia or no abnormalities is essential in the assessment of regression, all histological sections of the 170 biopsies and 170 cone biopsies were independently reviewed by two experienced pathologists (EG, JB). They were blinded for patient data and each othe?s diagnosis, and used p16 and Ki67 in their review to support their diagnoses in all cases. Regression was defined as a CIN2–3 in the cervical biopsy and CIN1 or less in the subsequent cone biopsy.

### Statistical Analysis

SPSS, version 18 (SPSS Inc., Chicago, IL, USA) was used for statistical analysis. Power calculation was based on a 25% difference between the current percentage regression probability and the expected regression probability. To obtain 90% power to detect this difference, assuming a type one error of 5%, 46 patients are needed. A drop-out rate of 30% due to histology ≤CIN1 and other exclusion criteria were taken into account.

Data are presented as median with range unless otherwise stated. The Kolmogorov-Smirnov test and visual inspection of plots were used to test for normal distribution. Comparisons of continuous variables were performed by 2-sided t-test or Mann-Whitney U-test, as appropriate. Comparisons of categorical variables were done using Chi-square. A multivariate binary logistic regression model and Cox regression model was applied to assess the influence of clinical variables as predictors for regression. Probabilities of 0.05 were considered as statistically significant.

## Results

The study population consisted of 170 women with a first-time onset of atypical cytological smear and histologically proven CIN2-3. No patient was lost to follow-up.

Basic and clinical characteristics of the study population are shown in table 1. Median age was 30.8 (25–41) years and the median biopsy-cone excision interval was 113 (84–171) days (7 cases had >140 days follow-up). The regression rate was 22% (38/170). There were 39 CIN2 and 131 CIN3 lesions with regression rates of 31% and 20%, respectively (p = 0.19). Age ≤15 years at first sexual intercourse was associated with a significantly lower regression rate (11%) compared to the group with a sexual debut after the age of 15 (26%, p = 0.04). Neither smoking, the number of lifetime sexual partners, nor the sexual activity span ( = interval between first sexual intercourse and age at study inclusion) differed significantly between the regression versus the non-regression group. 58% of the study population had given birth at the median age of 24 years. With nulliparous versus primiparous or multiparous, neither the number of children nor the maternal ages at first delivery were significantly correlated to the probability of regression (Table 1).

**Table 1:** Clinical characteristics of the study population according to regression or non-regression.

	Regression (n = 38)	Non-regression (n = 132)	p-value
Median age, years (range)	30.3 (25.1–41.0)	31.2 (25.0–41.0)	0.67
Median interval biopsy–cone excision, days (range)	113 (84–154)	113 (84–171)	0.22
Sexual activity span, years (range)	13.0 (6.1–25.4)	13.6 (6.0–25.6)	0.47
Age at first birth, years (range)	24.0 (18–38)	24.0 (16–32)	0.48
**Histology**			
CIN2	12 (31%)	27 (69%)	0.19
CIN3	26 (20%)	105 (80%)	
**Currently smoking**			
Yes	10 (18%)	45 (82%)	0.43
No	28 (24%)	87 (76%)	
**Number of lifetime sexual partners**			
1–4	8 (31%)	18 (69%)	0.31
≥5	30 (21%)	114 (79%)	
**Number of lifetime sexual partners**			
1–9	20 (25%)	60 (75%)	0.47
≥10	18 (20%)	72 (80%)	
**Age at first sexual intercourse, years**			
≤15	5 (11%)	39 (89%)	0.04
>15	33 (26%)	33 (26%)	
**Parity**			
Nulliparous	16 (26%)	45 (74%)	0.44
Primi/multiparous	22 (20%)	87 (80%)	
**Parity**			
0–2	35 (24%)	113 (76%)	0.41
3–4	3 (14%)	19 (86%)	
**Contraception**			
Non-hormonal	26 (29%)	65 (71%)	0.04
Hormonal	12 (15%)	67 (85%)	
**Consistent condom use**			
Yes	11 (55%)	9 (45%)	0.001
No	27 (18%)	123 (82%)	
**Condom use**			
Consistent	11 (55%)	9 (45%)	0.03
Inconsistent	3 (19%)	13 (81%)	
**Condom use**			
Inconsistent	3 (19%)	13 (81%)	0.9
No	24 (18%)	110 (82%)	

Contraception used during the study was divided into a non-hormonal group (20 consistent condom, 16 non-consistent condom, 1 pessary, 2 non-hormonal IUD, 52 none contraceptives) and a hormonal group (44 oral contraceptives, 27 hormonal IUD, 5 vaginal contraceptive ring, 2 contraceptive patch, 1 Depot Medroxyprogesterone Acetate injection). Only 20 of 170 patients (12%) reported consistent condom use. Clinicopathological features between the 20 consistent condom users and other patients did not differ significantly. Consistent condom use was associated with a significantly higher regression rate of 55% (hazard ratio 4.4) compared to patients using other types of contraception or non-consistent condom use (18%, p = 0.001). Inconsistent condom use did not significantly increase the regression rate (Table 1).

The non-hormonal contraception group had a regression rate of 29%, which was significantly higher than in the hormonal contraception group, 15% (p = 0.04). However, after adjusting for consistent condom use, the difference lost its significance.

In a univariate analysis consistent condom use (p<0.001), use of non-hormonal contraceptives (p = 0.04) and age at first sexual intercourse >15 years (p = 0.04) were significantly associated with regression. In a multivariate logistic regression model only consistent condom use remained as an independent predictor of regression.

Finally, a Cox regression analysis using the biopsy-cone interval as the independent variable, revealed condom use as the only independent regression predictor.

## Discussion

We evaluated the influence of different clinicopathological factors on regression of CIN2-3 in 170 patients. The overall regression rate was 22%, which is in line with previously reported regression rates of 5–50% [3], [4], [5]. Consistent condom use during the study period was associated with a significantly increased regression rate (55%), but inconsistent condom use was not. None of the other clinical factors added into multivariate analyses significantly predicted regression (Table 1).

Smoking, hormonal birth control and sexual behaviour as the number of lifetime sexual partners, the age at first sexual intercourse and the sexual activity span are considered risk factors for the development of CIN. However, only few studies have examined the influence of these factors on regression in patients with established CIN [5], [21], and none of them have evaluated the effect of condom use on regression. In agreement with these two studies, the current study confirmed that none of the examined clinical factors are independent predictors of CIN regression.

Hogewoning et al reported an increased chance of CIN2-3 regression in patients using condoms with a hazard ratio of 3.1 compared to non-condom users. The current study is not a hypothesis generating study, but an independent validation analysis confirming the results of Hogewoning et al. [18]. The comparable significantly increased regression rate among consistent condom users (hazard ratio 4.4) underlines that consistent condom use has a positive effect on regression of CIN2-3 lesions.

Three hypothetical explanations for the increased regression rate by consistent condom use can be discussed. First, the reduction of repetitively exposure of the cervical mucosa to HPV may strengthen the local immune system defence against the HPV infection and promote CIN regression. Secondly, the immunosuppressive effect of semen mediated by prostaglandins may play a role [22], [23]. This effect seems to be biologically meaningful for reproduction, but can diminish the patients defence against HPV in CIN2-3 lesions and potentially inhibit CIN regression [24]. A third intriguing hypothesis to explain the higher regression rate of consistent condom users may be that the latex of the condoms stimulates the general cellular immune response, which might be beneficial in the clearance of HPV.

Condom use as contraception was rather infrequent in the study population, and only 12% used condoms consistently. The current results, indicating that consistent condom use considerably increases the probability to regress and consequently reduces the risk for cone excision, could motivate a higher proportion of women to use this contraception type for a limited period of time. Motivation meetings including the patient? partners may also be important to increase compliance rate according consistent condom use.

The non-hormonal contraception group had a significantly higher regression rate than the hormonal contraception group. However, this difference was not longer significant after correcting for condom use. After excluding condom users, there was still no significant difference in regression rates between hormonal versus non-hormonal contraception users.

Previous studies have shown a decreasing regression rate with increasing CIN grade [4], [25]. In the current study we found the same trend, but the difference was not significant. The current study was underpowered to detect significant differences in CIN subgroups. Based on the observed 11% difference in regression rates, 654 first time onset CIN2-3 patients are needed to perform a study with adequate statistical power.

Smoking is a risk factor for CIN and invasive cancer development, and has been reported to be associated with decreased probability of regression [14], [26]. We found a lower, but not significant regression rate in smokers versus non-smokers (18% versus 24%). Two other studies on 100 and 125 patients came to the same conclusion [5], [18]. However, again the current study was probably underpowered to detect this difference, and with an alpha of 0.05, and a power of 0.80, one would need at least 400 patients.

It has been hypothesised that taking diagnostic biopsies may induce CIN regression by eliciting a local inflammatory response, which might influence the natural history on CIN. Further research concerning the biopsy-induced immune response is warranted.

The strengths of the current study are the prospective nature, the consecutive follow-up of all women with a first time onset CIN2-3 and the histological definition of CIN for the diagnosis and for CIN regression. Furthermore, two independent pathologists set the diagnoses, including the immunohistochemical biomarkers p16 and Ki67 as supporting analyses for CIN grading. Stavanger University Hospital is the only hospital in the region, making this a population-based study, which minimizes selection biases. Moreover, the patients’ age and interval between punch biopsies and cone excision was limited, which allows observing the natural history of CIN in a defined age group and time aspect.

A limitation of the study was the relatively short observational period between biopsy and cone excision as regression can take from months to years [4], [27], [28]. However, a longer observational period in the CIN2-3 patients would have been difficult to justify at the start of the study, as a longer interval with the subset histological diagnosis was regarded as to risky with respect to progression to cancer by the Ethical committee. Further, the sample size was rather moderate, which limits the separate interpretation of regression in some of the subgroups (i.e. smoking, CIN grade 2 or 3, CIN or cervical cancer in 1^st^ or 2^nd^ degree relatives).

In conclusion, this prospective study shows that consistent condom use over a median time period of 113 days increases the regression rate from 18% to 55%, while non-consistent condom use does not (19%). Based on the current data, consistent condom use for a limited time with thorough follow-up seems a promising intervention for younger women. It remains to be assessed if even higher regression rates can be achieved by longer consistent condom use exceeding the current study period.
